# Lovastatin-Enriched Rice Straw Enhances Biomass Quality and Suppresses Ruminal Methanogenesis

**DOI:** 10.1155/2013/397934

**Published:** 2013-01-15

**Authors:** Mohammad Faseleh Jahromi, Juan Boo Liang, Rosfarizan Mohamad, Yong Meng Goh, Parisa Shokryazdan, Yin Wan Ho

**Affiliations:** ^1^Institute of Tropical Agriculture, Universiti Putra Malaysia, 43400 Serdang, Selangor, Malaysia; ^2^Faculty of Biotechnology and Biomolecular Sciences, Universiti Putra Malaysia, 43400 Serdang, Selangor, Malaysia; ^3^Faculty of Veterinary Medicine, Universiti Putra Malaysia, 43400 Serdang, Selangor, Malaysia; ^4^Institute of Bioscience, Universiti Putra Malaysia, 43400 Serdang, Selangor, Malaysia

## Abstract

The primary objective of this study was to test the hypothesis that solid state fermentation (SSF) of agro-biomass (using rice straw as model); besides, breaking down its lignocellulose content to improve its nutritive values also produces lovastatin which could be used to suppress methanogenesis in the rumen ecosystem. Fermented rice straw (FRS) containing lovastatin after fermentation with *Aspergillus terreus* was used as substrate for growth study of rumen microorganisms using *in vitro* gas production method. In the first experiment, the extract from the FRS (FRSE) which contained lovastatin was evaluated for its efficacy for reduction in methane (CH_4_) production, microbial population, and activity in the rumen fluid. FRSE reduced total gas and CH_4_ productions (*P* < 0.01). It also reduced (*P* < 0.01) total methanogens population and increased the cellulolytic bacteria including *Ruminococcus albus*, *Fibrobacter succinogenes* (*P* < 0.01), and *Ruminococcus flavefaciens* (*P* < 0.05). Similarly, FRS reduced total gas and CH_4_ productions, methanogens population, but increased *in vitro* dry mater digestibility compared to the non-fermented rice straw. Lovastatin in the FRSE and the FRS significantly increased the expression of HMG-CoA reductase gene that produces HMG-CoA reductase, a key enzyme for cell membrane production in methanogenic Archaea.

## 1. Introduction

Over the last 250 years, the concentration of atmospheric methane (CH_4_) increased by approximately 150% [1], with agricultural activities contributing 40% of the total anthropogenic source, of which 15 to 20% is from enteric fermentation in ruminants [2]. On the other hand, ruminal CH_4_ production accounts for between 2 and 15% of dietary energy loss for the host animals [3]. Because of its negative effect on environment and the host animal nutrition, mitigation of enteric CH_4_ fermentation in ruminant livestock, including the use of various mitigating agents, such as ionophores [4], organic acids [5], fatty acids [6], methyl coenzyme M reductase inhibitors [7], and oil [8] has been extensively researched. However, these technologies have limited application primarily because besides suppressing CH_4_ they also suppressed nutrients digestibility and thus overall animal productivity. 

Rice straw (RS) is one of the most abundant agricultural by-products, with nearly 90% of the world annual production in Asia [9]. The traditional method for disposing bulk of the RS after grain harvest is by burning [10] resulting in environmental pollution. On the other hand, ruminant animals can convert this fiber-rich biomass into high-quality animal protein (i.e., meat and milk) for human consumption. However, this highly efficient biological conversion of biomass into human food needs to be balanced against the concomitant production of CH_4_ which has often been implicated as source of greenhouse gases affecting global warming. Biological treatment has been shown to be able to hydrolyse the macromolecules of the lignocelluloses into usable nutrients and thus improved the quality of agricultural biomass as ruminant feed [11]. 

Lovastatin (C_24_H_36_O_5_, M.W. 404.55) is a secondary product of idiophase (secondary phase) of growth of fungi [19] and is an inhibitor of enzyme 3-hydroxy-3-ethylglutaryl coenzyme A (HMG-CoA) reductase [EC 1.1.1.34], a key enzyme in cholesterol production pathway in humans [20]. There is a similarity between cholesterol formation in human and cell membrane formation in the Archaea as the lipid side of phospholipids in the cell membrane of Archaea is isoprenoid chains [21]. Isoprenoid formation is an intermediate step of cholesterol production pathway (Mevalonate pathway) and HMG-CoA reductase is also a key enzyme for its production [22]. Therefore, as an inhibitor of HMG-CoA reductase, lovastatin suppresses isoprenoid production and thus cholesterol synthesis and membrane formation in the Archaea. Wolin and Miller [23] showed that lovastatin significantly reduced growth and activity of pure methanogenic bacteria without any negative effect on cellulolytic bacteria. In their study, pure statin was added to the broth medium of individual bacteria without examining the effects of statin on the function of mixed rumen microorganisms. Moreover, pure statin is too expensive to be used as feed additive and thus has limited application for mitigation of CH_4_ in ruminants. 

We have previously reported that *Aspergillus terreus *can be used to produce lovastatin in solid state fermentation (SSF) using RS as substrate with maximum production of 260.8 mg/kg DM lovastatin obtained after 8 days of fermentation [24]. The main objective of this study was to test the hypothesis that fungal treatment of agricultural biomass, using RS as model, can improve its nutritive value and in addition, as an agent for mitigation of CH_4_ without negatively affecting nutrient digestibility in the rumen ecosystem. To achieve the above, RS was fermented in SSF using *Aspergillus terreus *(ATCC74135) to produce lovastatin and to reduce its lignocelluloses content. Methanolic extract from the FRS containing lovastatin was evaluated for reduction of methanogenesis in rumen fluid using *in vitro* gas production technique and finally the potential of the fermented rice straw (FRS) as inhibitor of methanogenesis and efficiency of microbial degradability of the substrate were evaluated.

## 2. Materials and Methods

### 2.1. Substrate, Microorganism, and Spore Suspension

Fermented rice straw was prepared in SSF using *A. terreus* ATCC 74135 according to the method described previously [24]. The concentration of lovastatin in the FRS was 260.8 mg/kg DM after 8 days of fermentation [24] and the dried FRS (60°C for 48 h) was used in the present study.

### 2.2. Transmission Electron Microscopy (TEM)

The procedure of sample preparation by Hayat [25] with minor modified by the Electron Microscopy Unit, Institute of Bioscience, Universiti Putra Malaysia, was used for the TEM study. The RS and FRS samples were cut into 1 mm and put in separated vials in triplicate and fixed in fixative solution (4% glutaraldehyde) for 2 days at 4°C. In the next step, samples were washed with 0.1 M sodium cacodylate buffer for 3 changes of 30 min each. For postfixation, samples were kept in 1% osmium tetroxide for 2 h at 4°C and then washed again with 0.1 M sodium cacodylate buffer for 3 changes of 30 min each. Samples were kept in different concentrations of acetone (35, 50, 75 and 95%) for 30 min each and finally in 100% acetone for 3 times of 1 h to dehydrate. For infiltration of the specimen, samples were dissolved into acetone-resin (1 : 1) for 4 h, acetone-resin (1 : 3) for overnight, 100% resin over night, and finally 100% resin for 4 h. In the next step, samples were put in beam capsules and filled up with fresh resin and kept in oven at 60°C for 48 h for polymerization. Glass knife and ultramicrotome were used to cut the samples into 1 *μ*M and samples placed onto glass slides, stained with toluidine blue, dried, washed the stain, and examined under light microscope. After selecting the area of interest, the sections were stained with uranyl acetate for 15 min and washed by distilled water for 3 times. Transmission electron microscopy observations were carried out using Transmission Electron Microscope (Hitachi H-7100, Japan).

### 2.3. Preparation of Methanolic Extract

For preparation of methanolic extract, 200 g of the FRS were mixed with 1.5 L of methanol and shacked for 2 h at room temperature. The solid sample was removed from the suspension using 0.45 *μ*M vacuum filter. Methanol from extract was removed by evaporation at 45°C using rotary evaporator (Eppendorf, USA). The concentration of lovastatin in the FRS extract was quantified using HPLC according to the method that described previously [24].

### 2.4. *In Vitro* Gas Production

Gas production was determined by the procedure described by Menke and Steingass [26]. For studying the effect of fermentation process on rumen microorganisms, two experiments of *in vitro* gas production were designed. 

*Experiment  1*. The aim of this experiment was to examine the effect of fermented rice straw extract (FRSE) containing lovastatin on rumen fermentation, microbial activity, and population. In this study, 500 mg of ground RS were transferred in 100 mL glass syringes (Haberle Labortechnik, Germany) followed by the addition of 200 *μ*L of methanol (control) while in the treatments, 10 mg (treatment 1) and 20 mg (treatment 2) of dry FRSE were dissolved in the 200 *μ*L methanol before the latter was added in the glass syringe contain 500 mg RS.

*Experiment  2*. In this experiment, the FRS (containing lovastatin) was compared with RS to investigate their effect on microbial activity in the rumen ecosystem. 500 mg of RS and FRS were transferred into 100 mL calibrated glass syringes (Haberle Labortechnik, Germany) for the *in vitro* gas production study and their effect on rumen microflora activity and population. 

All treatments were replicated three times and repeated in two separate runs. Buffer and mineral solution [26] was prepared and placed in a 39°C water bath under continuous flushing with CO_2_. Rumen fluid was collected before the morning feeding from two rumen-fistulated steers fed an equal weight mixture of 40% concentrate and 60% grass hay twice daily at 0800 and 1800 h. Rumen fluid was collected from the rumen with a manually operated suction pump and transferred into two prewarmed bottle, filtered through eight layers of cheesecloth, and flushed with CO_2_. Rumen fluid (800 mL) was added to the buffered mineral solution (1600 mL) with constant stirring, while maintained in a water bath at 39°C. About 30 mL of buffered rumen fluid was transferred into syringes containing each treatment. The above procedures were conducted under continuous flushing with CO_2_. After closing the clips on the silicon tube attached to the syringe tip, syringes were gently shaken and the clips were opened to remove the gas by pushing the piston upwards to achieve complete gas removal. The clip was closed, the initial volume recorded, and the syringe was placed in the water bath incubator at 39°C for 48 h. Standard hay (University of Hohenheim, Stuttgart, Germany) with an estimated gas production of 49.61 mL/g DM was used as a standard to calibrate the *in vitro* gas production system. Gas production was recorded at 2-hour intervals and at the end of the incubation the liquid layer of each syringe was sampled for pH, volatile fatty acids analysis, bacterial quantification, and gene expression. *In vitro* dry matter digestibility (IVDMD) was determined according to Tilley and Terry [27].

### 2.5. Methane and Hydrogen Determination

The concentrations of CH_4_ and H_2_ in the headspace gas phase of syringes were determined by injecting 500 *μ*L of the gas from each sample to the gas chromatography (Agilent 6890 Series Gas Chromatograph, Wilmington, DE, USA). Separation of the gases was achieved using a HP-Plot Q column (30 m × 0.53 mm × 40 *μ*M) (Agilent Technologies, Wilmington, DE, USA). Nitrogen was used as carrier gas with flow rate of 3.5 mL/min (MOX, Kuala Lumpur, Malaysia). The isothermal oven temperature was 50°C and the separated gases were detected using thermal conductivity detector in 4 min of run time. Calibration was completed using standard gas prepared by Scott Specialty Gases (Supelco, Bellefonte, PA, USA) which contain 1% of CH_4_, CO, CO_2_, O_2_, and H_2_.

### 2.6. Volatile Fatty Acids Determination

After incubation, samples were centrifuged at 1340 ×g for 10 min and 3 mL of the supernatant fluid were transferred to 15 mL centrifuged tube and 600 *μ*L of 24% metaphosphoric acid were added to acidify the samples and allowing the volatile fatty acids (VFA) to be vaporized in the gas chromatography injection port and the samples were kept for 24 h at room temperature. The samples were then centrifuged (1340 ×g for 20 min) and 0.5 mL of supernatant plus 0.5 mL of internal standard (20 mmol, 4-methylvaleric acid) were transferred into 2 mL glass tube and kept at 4°C pending for analyses. The concentrations of VFA were determined by gas chromatography (Agilent Technologies, USA, Model GC6890) with a flame ionization detector (FID) and fused silica capillary column. Nitrogen was used as carrier gas. Acetate (20 mmol), propionate (10 mmol), Butyrate (10 mmol), isobutyrate (10 mmol), valerate (10 mmol), and isovalerate (10 mmol) were used as standard solution [28].

### 2.7. DNA Extraction and Quantitative Real-Time PCR

One and half millilitre (1.5 mL) of rumen fluid sample was used for microbial quantification by real time PCR. DNA was extracted from rumen fluid using the QIA amp DNA Stool Mini Kit (Qiagen Inc., Valencia, CA, USA) according to the manufacturer's protocol. The extracted DNA was stored at −20°C until used. The DNA for each group of microorganisms was amplified from the DNA extract of rumen fluid using specific primers as indicated in Table 1. The PCR reaction was performed on a total volume of 100 *μ*L using the i-Taq TM DNA Polymer ASE kit (INTRON Biotechnology, Korea). Each reaction included 2.5 *μ*L i-Taq DNA Polymer ASE (5 U/ *μ*L), 10 *μ*L PCR buffer, 5 *μ*L of each Primer (10 pM), 10 *μ*L dNTP (2.5 *μ*M each), 5 *μ*L of DNA sample of rumen fluid, and 62.5 *μ*L H_2_O. Purified PCR products were cloned into the pCR 2.1 TOPO vector using PCR 2.1 TOPO TA Cloning Kit (Invitrogen Ltd., USA) according to the protocol of manufacturer. Produced plasmid DNAs were sequenced for confirmation. Plasmid DNA from each group of microorganisms was used for preparation of standard curve and the purity and concentration of Plasmid DNA in each sample were measured using a spectrophotometer and the number of copies of a template DNA per mL of elution buffer was calculated using the formula that is available online (http://www.uri.edu/research/gsc/resources/cndna.html):

(1) number  of  copeis  =Amount  of  DNA  (μg/mL)×6.022×1023Length  (bp)×109×650.

Standard curves were constructed using serial dilution of plasmid DNA of each microbial group.

Primers used to quantify the population of different groups of microorganisms are shown in Table 1. Real-time PCR was performed with the BioRad CFX96 Touch (BioRad, USA) using optical grade plates. The PCR reaction was performed on a total volume of 25 *μ*L using the iQTMSYBR Green Supermix (BioRad, USA). Each reaction included 12.5 *μ*L SYBR Green Supermix, 1 *μ*L of each Primer, 1 *μ*L of DNA samples, and 9.5 *μ*L H_2_O. 

The following reaction conditions were applied to each well: an initial 5-min incubation at 94°C; and 40 cycles of denaturation at 94°C for 20 s, annealing (temperatures for different primers described in Table 1) for 30 s, and extending at 72°C for 20 s. To confirm the specificity of amplification, melting curve analysis was carried out after the last cycle of each amplification and PCR products were verified on a 2% (W/V) agarose gel that runs for 40 min at 80 V. The expected sizes of amplified fragments were presented in Table 1. The amplification efficiency was calculated using the equation *E* = (10^−1/slope^ − 1) × 100%, and only the data generated from reactions with efficiency between 90 and 110% were used for further analysis [29].

### 2.8. RNA Extraction and Gene Expression

At the end of 48 h *in vitro* gas production, 1.5 mL of rumen liquor was collected and stored in −80°C for microbial RNA extraction. RNA was extracted using Ribo Pure Bacteria RNA Isolation kit (AMBION, Austin, TX, USA, AM1925) according to the manufacturer's protocol. Two-step method was used for determination of relative gene expression. RNA samples were reverse transcribed into First-strand cDNA using First-Strand cDNA synthesis Kit according to the manufacturer's instructions (Maxime RT-PCR Kit, iNtRON). In the next step, real-time PCR was performed with the BioRad CFX96 Touch (BioRad, USA) using optical grade plates. The PCR reaction was performed on a total volume of 25 *μ*L using the iQTMSYBR Green Supermix (BioRad, USA). Each reaction included 12.5 *μ*L SYBR Green Supermix, 1 *μ*L of each Primer, 1 *μ*L of cDNA samples, and 9.5 *μ*L H_2_O. The primer that was used for amplification of methyl coenzyme-M reductase subunit A (*mcrA*) gene in the terminal step of the methanogenesis pathway and HMG-CoA Reductase (*hmg*) gene is shown in Table 1. 16S rRNA was used as reference gene [18]. The 2^−ΔΔCt^ method was used for expression analysis of the *mcrA* and *hmg* genes [30].

### 2.9. Statistical Analysis

All experiments were conducted with 6 replicates per treatment. Individual culture syringes were considered as experimental units. In the first experiment (effect of FRSE on rumen microorganisms), data were analyzed as a completely randomized design (CRD) using the general linear model (GLM) procedure of SAS 9.2 [31]. All multiple comparisons among means were performed using Duncan's new multiple-range test (*P* < 0.05). In the second experiment (effect of FRS and RS on rumen microoorganisms), *t*-test method of SAS 9.2 [31] was used for statistical analysis.

## 3. Results

### 3.1. Lovastatin Production

**Figure 1:**
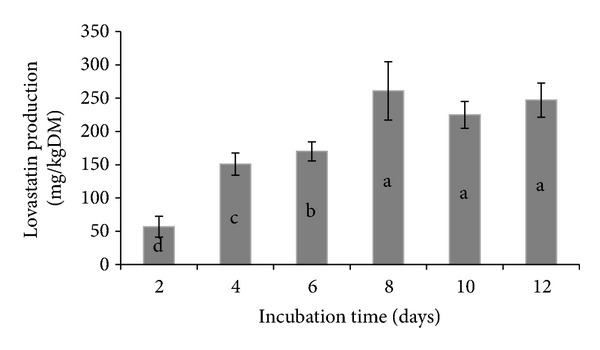
Lovastatin production by *A. terreus* in solid-state fermentation at different incubation times. a, b, c, and d: indicating means that differed significantly [24].

Lovastatin productions by *A. terreus* using rice straw as substrate in SSF at different incubation times as quantified by HPLC are shown in Figure 1 as we previously reported [24]. Since maximum production of the lovastatin was detected after 8 d of fermentation with maximum production of 260.8 mg/kg DM, a sample from the above treatment was selected to examine the effect of FRS containing lovastatin on rumen microorganisms. On the other hand, concentration of lovastatin in FRSE was 97 mg/g DM. 

### 3.2. Lignocellulose Reduction

**Figure 2:**
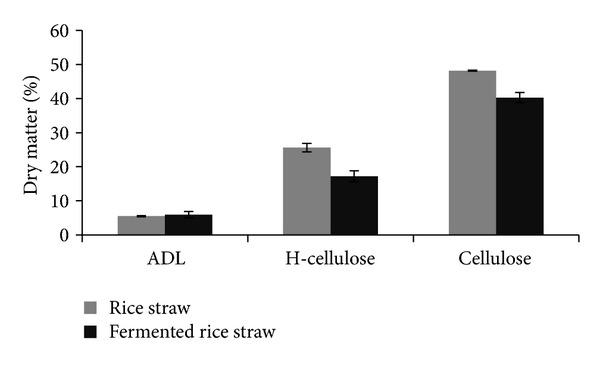
Effect of biological treatment using *A. terreus* on lignocellulose composition of rice straw (% dry matter).

The abilities of *A. terreus* to reduce lignocelluloses content of rice straw are shown in Figure 2. SSF significantly (*P* < 0.01) reduces cellulose and hemicelluloses contents but not lignin (ADL) of the RS. Hemicellulose was reduced by 32.68%, (from 25.62 to 17.20%) while cellulose was reduced by 16.32% (from 48.17 to 40.31%) after 8 d of fermentation.

**Figure 3:**
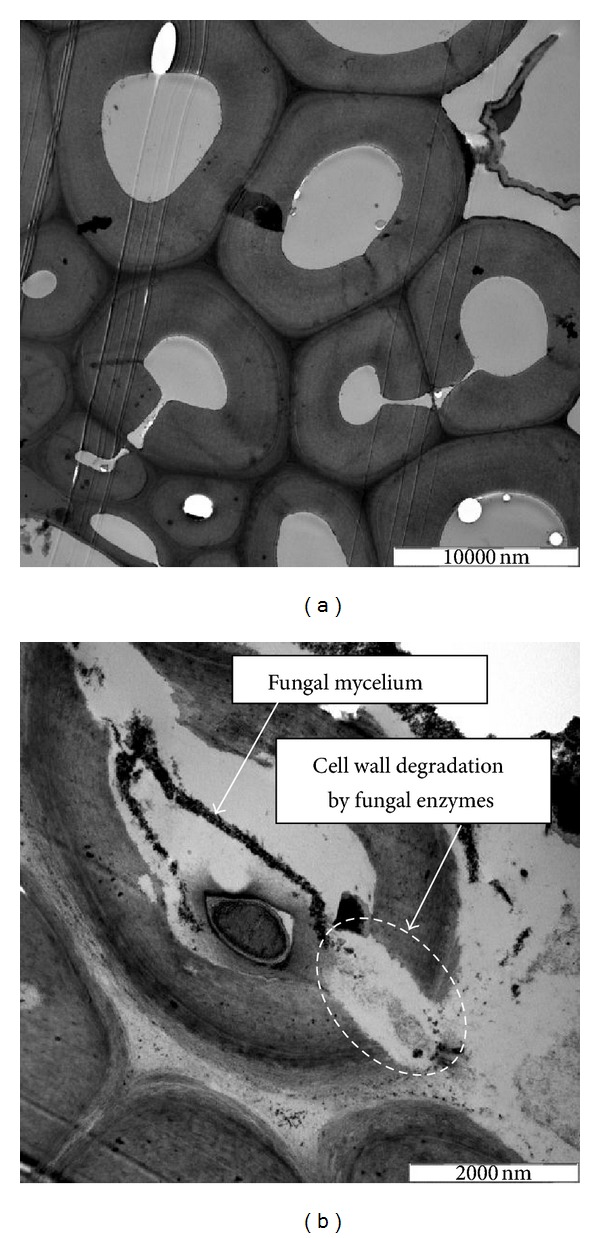
Effect of *A. terreus* on cell wall structure of rice straw; (a) before and (b) after fermentation.

Transmission electron micrograph (Figure 3) clearly shows the ability of *A. terreus* to break down the lignocellulose of FRS and thus increased its surface areas for ruminal microorganisms, specially cellulolytic bacteria to adhere and degradate them further.

### 3.3. Effect of FRSE on Rumen Microorganisms

One of the main objectives of this study was to provide evidence that fermentation, besides degrading the lignocellulose content of the FRS (which containing lovastatin), also reduces ruminal methanogenesis. To achieve the above-mentioned objective, lovastatin from FRS was extracted using methanol and its quantity in the crude extract was determined using HPLC. The lovastatin content of the FRSE was 97 mg/g dry matter of the crude extract. 

The FRSE containing lovastatin at 10 and 20 mg levels significantly reduced the total *in vitro* gas production by mixed rumen microorganisms after 12 h (*P* < 0.05), 24, 36, and 48 h (*P* < 0.01) incubation (Table 2). Total gas production after 48 h incubation was 74.4 mL for the control and 71 and 66.6 mL for 10 and 20 mg of FRSE, respectively. The two levels of FRSE also significantly (*P* < 0.01) reduced total CH_4_ production by rumen methanogenic Archaea after 48 h incubation (723.21, 616.62, and 521.65 *μ*M for the control, 10 mg, and 20 mg FRSE, resp.). The FRSE also reduced (*P* < 0.01) the rate of gas and CH_4_ production (mL/h) as well as the ratio of CH_4_ to total gas (Table 2). 

The effects of FRSE on VFA production by rumen microorganisms are shown in Table 3. The FRSE treatments increased VFA, particularly acetate production (*P* < 0.01). However, it has no effect (*P* > 0.05) on IVDMD of the rice straw and pH of the rumen fluid after 48 h incubation. Rates of gas and CH_4_ productions per unit of VFA produced in the FRSE treatments were lower (*P* < 0.01) than those for the control. 

### 3.4. Effect of FRSE on Microbial Population

The effect of FRSE on rumen microbial population is presented in Table 4. Population of total methanogens in the treatments containing FRSE was lower (*P* < 0.01) than the control. FRSE also reduced (*P* < 0.05) the population of Methanobacteriales species (the dominant group of methanogenic Archaea in the rumen) and anaerobic fungi (*P* < 0.01) in the rumen liquid. Although treatment containing 20 mg FRSE reduced (*P* < 0.05) the population of total bacteria, the population of cellulolytic bacteria including *Ruminococcus albus, Fibrobacter succinogenes* (*P* < 0.01), *and Ruminococcus flavefaciens* (*P* < 0.05) that play important roles on the degradation of lignocellulosic materials increased. FRSE has no significant effect on population of Protozoa (*P* > 0.05).

### 3.5. Effect of FRS on Rumen Microorganisms

Total gas production after 2, 4, and 8 h incubation was higher (*P* < 0.01) in the FRS treatments but thereafter the corresponding values were lower for FRS (Table 5). FRS also reduced (*P* < 0.01) the total CH_4_ production, rates of total gas, and CH_4_ production by rumen microorganisms after 48 h incubation. The ratio of CH_4_ to total gas in the treatment containing FRS was lower (*P* < 0.05) than non-fermented RS. In addition, quantity of hydrogen (H_2_) in the FRS was lower (*P* < 0.05) than RS treatment.

Effects of FRS on VFA production by rumen microorganisms are presented in Table 6. Although FRS has no effect on total VFA production (*P* > 0.05) it increased IVDMD, ratio of total gas : VFA, and CH_4_ : VFA (*P* < 0.01). 

Similar to the FRSE, FRS reduced the population of total methanogens, Methanobacteriales (*P* < 0.05), total fungi, total bacteria, and *Fibrobacter succinogenes* (*P* < 0.01), but increased the population of *Ruminococcus albus* (*P* < 0.01) (Table 7). FRS has no effect on the population of *Ruminococcus flavefaciens* and protozoa (*P* > 0.05).

### 3.6. Expression of *mcrA* and *hmg* Genes

**Figure 4:**
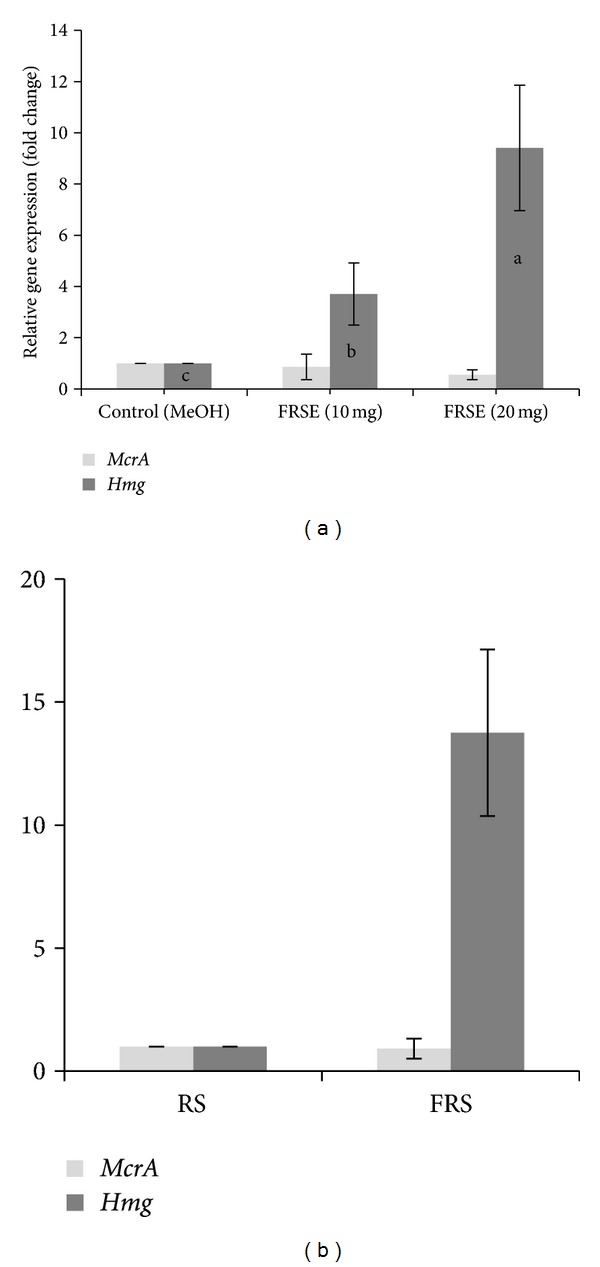
Effect of fermented rice straw extract (FRSE) (Figure 4(a)) and fermented rice straw (FRS) (Figure 4(b)) on expression of *mcrA* and *hmg* genes in the rumen liquid sample. Treatment has no significant effect on expression of *mcrA* gene (*P* > 0.05) and significantly increased the expression of *hmg* gene (*P* < 0.01). a, b, and c: indicating differences among means between samples.

The effects of FRSE and FRS on expression of *mcrA* and *hmg* genes of methanogens bacteria in the rumen liquid samples are presented in Figure 4. After 48 h incubation, both FRSE (Figure 4(a)) and FRS (Figure 4(b)) significantly increased the expression of *hmg* gene compared to the control (*P* < 0.01) but had no effect on *mcrA* gene.

## 4. Discussion

### 4.1. Lovastatin Production and Lignocellulose Reduction

Solid-state fermentation is the growth of microorganisms on moist solid materials in the absence or near absence of free water [32] and the United State Food and Administration (USFAD) has approved its use for commercial production of clinical drugs, including lovastatin from fungi [33]. Although pure lovastatin has been shown to significantly suppress methanogenesis [34], it is too expensive to be used as an additive in ruminant diets for CH_4_ mitigation. We [24] have previously produced lovastatin by fermenting rice straw, an agrobiomass, using *A. terreus*. The optimal concentration of lovastatin obtained after 8 days fermentation using the above procedure was approximately 261 mg/g DM, which is much lower than the 4 to 6 mg lovastatin produced from per g of energy-rich rice grain as substrate [35]. However, based on results of the fiber reduction (Table 2) and effectiveness of the suppression of CH_4_ emission (Tables 2 and 4), the lovastatin content in the FRS is believed to be sufficient for the intended purpose in this study.

The ability of *A. terreus* to produce cellulolytic enzymes has been well documented [36–39] with xylanase as the main enzyme in SSF by *A. terreus *[37]. The higher reduction of hemicelluloses (from 25.62 to 14.94%) which constitute primarily xylan, compared to cellulose (from 48.17 to 38.36%) over the control in our study, reaffirmed the above. High lignocellulose content in most agro-biomass, including RS, is the main constraint for its widespread use as ruminant feed. Biological treatment has been suggested to be able to improve the quality of these materials [40] as the polysaccharides including cellulose and hemicelluloses are converted into monomers such as glucose and xylose, and the latter are used for production of more fungal cell mass. Therefore, the reduction in the lignocelluloses content in the FRS in this study is an indication of an improvement of the fermented material. The ability of *A. terreus *to use the lignocelluloses materials for growth and to increase it cell mass on the surface of rice straw and to breakdown its lignocellulosic structure of the FRS is clearly shown in Figures 2 and 3, respectively. A previous report [40] showed that fermented agrobiomass containing fungal cell mass has higher digestibility compared to non-fermented materials. 

### 4.2. Effect of FRS and FRSE on Rumen Microbiota

Rumen contains an array of microorganisms playing their respective roles in the degradation of fiber component of feed materials [41–43]. Volatile fatty acids production from the rumen microbial activity and the subsequent microbial mass produced can be digested and absorbed by the host animals for growth and other functions [41]. The above are the beneficial effect of ruminal microbial activity. In contrast, rumen methanogenic Archaea are microorganisms that result in losses of dietary energy by converting H_2_, carbon, and VFA (mainly acetate) into CH_4_ in the process of methanogenesis. To overcome this negative nutritional effect on the host animals, together with the role of CH_4_ as a greenhouse gas on global warming and climatic change, many CH_4_ mitigation agents have been tested to inhibit methanogenesis in the rumen. However, most of the existing methodologies are not applicable under farm conditions, primarily because the inhibitors are also suppressing activity of cellulolytic bacteria and thus reduced fiber digestion. 

There is a close similarity in cholesterol biosynthesis in the eukaryotic cells and cell membrane biosynthesis in the Archaea. HMG-CoA reductase is a key enzyme that catalyses the production of mevalonic acid from HMG-CoA in the eukaryotes and Archaea, and statins are the inhibitor of this enzyme. This enzyme is essential for production of Geranylgeranyl isopentenyl-5-pyrophosphate for synthesis of the branched isoprene side chains in the Archaeal phospholipids. The main difference between Archaea and other microorganisms is the structure of cell membrane; the lipid side of phospholipid in the Archaea is branched isoprene side chains but the lipid of phospholipid in other microorganisms is fatty acid [22]. Methanogenic bacteria are the main group of Archaea in the rumen [23]; thus, they will be the key microorganisms (except for fungi, see later discussion) affected by any HMG-CoA reductase inhibitors within the rumen. Our results support the above hypothesis. Both the lovastatin-rich FRSE and FRS reduced the population of methanogenic Archaea (Tables 4 and 7) and CH_4_ production in the rumen fluid cultures (Tables 2 and 5). There was a decrease in the population of total bacteria but the population of *Ruminococcus albus*, one of the most important cellulolytic bacteria, was significantly increased in both the FRSE and FRS treatments compared to the control (Tables 4 and 7). Although FRSE significantly increased VFA production (Table 3) compared to the control, similar increment was not shown when FRS was used (Table 6). The significant reduction in the total gas and CH_4_ productions (Table 5) without affecting the VFA production is an indication that lovastatin could suppress CH_4_ without negatively affecting microbial degradation efficiency in the FRS rumen fluid. In both, the FRSE and FRS experiments, the ratio of total gas/VFA and CH_4_/VFA was significantly reduced, indicating that the inhibitive effect of lovastatin on CH_4_ production was absolute and not a relative reduction due to the suppression of the total gas production.

The higher apparent IVDMD and acetate production accompanied by lower gas and CH_4_ productions and no differences in VFA production in the FRS treatment compared to the control (unfermented rice straw) (Table 6) seems to be difficult to reconcile biologically. One possible explanation to the above phenomenon is because FRS contained higher soluble materials, such as fungal biomass and soluble sugars [40] and part of this material could have escaped the fermentation process and was later hydrolyzed in the pepsin/HCl solution treatment during the determination of the apparent IVDMD. 

Another possibility for the above phenomenon is alterations of the cellulolytic bacteria and fungi populations in the FRS and FRSE treatments. Our results show that although the population of total bacteria was reduced, *R. albus*, one of the most important cellulolytic bacteria, was significantly increased in the FRS (and FRSE) treatments (Tables 4 and 7). Miller and Wolin [44] reported that in cellulose substrate, *R. albus *produces high quantity of acetate but very little (unquantifiable) amount of gas after 32 h incubation. We believe that the increased population of the acetate but not gas producing *R. albus* (more than 10^8^ cell/mL), compared to the other two groups of cellulolytic bacteria (*F. succinogenes*, 10^7^ cell/mL; *R. floriu*, 10^6^ cell/mL), is partially responsible for the low production of the total gas and CH_4_ without affecting IVDMD and VFA production in the FRS treatment.

In addition, both experiments (FRSE and FRS) showed a significant reduction in the population of the anaerobic fungi in the rumen fluid culture (Tables 4 and 7). Both, synthetic and fermented statins have been reported [45–47] to exhibit antifungal activites, including lovastatin on the activity of *Zygomycetes* and *Rhizomucor* species under *in vitro* condition [45]. Since fungi are eukaryotic microorganisms, HMG-CoA reductase is present in them [48]. Therefore, lovastatin has the same inhibitive effect on the HMG-CoA reductase enzyme in fungi as it has in methanogenic Archaea. According to Pearce and Bauchop [49], rumen fungus (*Neocallimastix frontalis*) produces high quantity of H_2_ in cellulose substrate (approximately 44% of the total gas). Similarly, Yarlett et al. [50] reported that pure culture of rumen fungi produced more H_2_ than in a mixed culture of rumen fungi and methanogenic Archaea. The above information highlights that rumen fungi is an important source of H_2_ for the rumen methanogenic Archaea. Furthermore, Bernalier et al. [51] reported that rumen fungi (*Neocallimastix frontalis, Piromyces communis*, and *Caecomyces communis*) produced 7 to 10 times higher formate than cellulolytic bacteria (*R. flavefaciens* and *F. succinogenes*) in the same cellulose substrate. Since H_2_ and formate are the main substrates for the production of CH_4_ by methanogens in the rumen, the reduction of the fungi population in the FRS and FRSE treatments could also contribute to the reduction in CH_4_ production and without causing an accumulation of H_2_ in the rumen ecosystem.

We would like to propose that the increased *R. albus *(high acetate and low gas producing cellulolytic bacteria), decreased fungi (low acetate and high H_2_ producers) population as affected by lovastatin, and the increased digestibility of the FRS are the contributing factors to the high IVDMD and acetate production but lower gas and CH_4_ productions in the FRS treatment. 

### 4.3. Gene Expression

Results of the gene expression studies showed that lovastatin significantly increased the expression of *hmg* gene but not that of *mcrA* gene (producer of enzyme in the last step of methanogenesis pathway) (Figure 4). Our finding suggests that the controlling factor for the upward expression of the *hmg* gene is the concentration of mevalonic acid produced by HMG-CoA and catalyzed by the HMG-CoA reductase. Inhibition of the HMG-CoA reductase by lovastatin suppresses mevalonic acid production, and the reduced mevalonic acid concentration signalled to increase the expression of *hmg* gene and production of higher mRNA and enzyme in the rumen methanogens. Enhancement of the relative expression of genes involved in the process of cholestrol biosynthesis by lovastatin was reported previously [52]. 

## 5. Conclusion

Lovastatin can be produced in SSF using *A. terreus* and rice straw as substrates as we previously reported [24]. The present study showed that *A. terreus* has the potential to break down lignocelluloses, particularly hemicelluloses in the rice straw, and improved the quality of this agro-biomass as ruminant feed. The above suggestion is supported by the higher IVDMD and acetate production, suggesting higher microbial activity in the FRS treatment compared to the untreated rice straw. Lovastatin in the FRS and FRSE significantly reduced CH_4_ production and methanogens population, indicating that SSF of rice straw using *A. terreus* is an effective method to enhance the quality of this biomass and at the same time provides a practical method to mitigate methanogenesis and thus enteric CH_4_ production in ruminants. Since lovastatin also has the potential for cholesterol reduction through its inhibitory effect on HMG-CoA reductase and antioxidant activity [53, 54], feeding the fermented rice straw could potentially produce lower cholesterol and high-quality animal products. 

## Figures and Tables

**Table 1:** Names, sequences, application, product size, annealing temperature and references of the primers used.

Target group	Sequence 5′–3′	Application	Product size (bp)	Annealing temperature (°C)	Reference
Total bacteria F	CGGCAACGAGCGCAACCC	Quantification	145	55	[12]
Total bacteria R	CCATTGTAGCACGTGTGTAGCC				

Total fungi F	GAGGAAGTAAAAGTCGTAACAAGGTTTC	Quantification	121	55	[13]
Total fungi R	CAAATTCACAAAGGGTAGGATGATT				

Methanobacteriales F (MBT857F)	CGWAGGGAAGCTGTTAAGT	Quantification	343	58	[14]
Methanobacteriales R (MBT1196R)	TACCGTCGTCCACTCCTT				

*Ruminococcus albus* F	CCCTAA AAGCAGTCTTAGTTCG	Quantification	175	55	[12]
*Ruminococcus albus* R	CCTCCTTGCGGTTAGAACA				

*Ruminococcus flavefaciens* F (Rf154f)	TCTGGAAACGGATGGTA	Quantification	259	60	[12]
*Ruminococcus flavefaciens* R (Rf425r)	CCTTTAAGACAGGAGTTTACAA				

*Fibrobacter succinogenes* F	GTTCGGAATTACTGGGCGTAAA	Quantification	122	55	[13]
*Fibrobacter succinogenes* R	CGCCTGCCCCTGAACTATC				

Protozoa F	CTTGCCCTCYAATCGTWCT	Quantification	223	55	[15]
Protozoa R	GCTTTCGWTGGTAGTGTATT				

Methanogens (*mcrA*)-F	TTCGGTGGATCDCARAGRGC	Quantification gene expression	140	58	[16]
Methanogens (*mcrA*)-R	GBARGTCGWAWCCGTAGAATCC				

HMG-CoA reductase R	GGCTGTGAATTACCGCATATGG	Gene expression	117	55	[17]
HMG-CoA reductase F	TAACGGTCCGGCTACACCTACA				

16S rRNA F	CCGGAGATGGAACCTGAGAC	Gene expression (reference gene)	160	55	[18]
16S rRNA R	CGGTCTTGCCCAGCTCTTATTC				

**Table 2:** Effect of fermented rice extract (FRSE) on *in vitro* gas, methane and hydrogen production and rate of gas production.

Gas production (mL)	Ethanol (control)	FRSE (10 mg)	FRSE (20 mg)	Significant
2 h	2.7 ± 0.4	2.8 ± 0.5	3.0 ± 0.3	NS
4 h	4.7 ± 0.7	4.9 ± 0.8	5.3 ± 0.4	NS
8 h	6.9 ± 0.9	6.8 ± 1.1	7.3 ± 0.5	NS
12 h	12.8 ± 1.0^a^	11.0 ± 1.3^b^	11.2 ± 0.3^b^	*
24 h	38.6 ± 2.1^a^	31.8 ± 1.4^b^	28.7 ± 0.9^c^	**
36 h	58.3 ± 2.1^a^	53.0 ± 1.4^b^	46.5 ± 2.8^c^	**
48 h	74.4 ± 2.1^a^	71.0 ± 2.0^b^	66.6 ± 1.3^c^	**
Methane production				
%	21.76 ± 1.16^a^	19.47 ± 1.50^b^	17.55 ± 0.80^c^	**
*μ*M	723.21 ± 52.56^a^	616.62 ± 41.38^b^	521.65 ± 25.21^c^	**
Hydrogen production				
%	5.65 ± 0.88	5.33 ± 0.80	4.79 ± 1.21	NS
*μ*M	187.22 ± 9.189	153.46 ± 11.255	154.72 ± 12.996	NS
Rate of GP (mL/h)	1.55 ± 0.04^a^	1.48 ± 0.04^b^	1.39 ± 0.03^c^	**
Rate of CH_4_ (mL/h)	0.34 ± 0.02^a^	0.29 ± 0.02^b^	0.24 ± 0.01^c^	**
CH_4_/total	0.22 ± 0.012^a^	0.20 ± 0.015^b^	0.18 ± 0.008^c^	**

Data are mean ± SD.

NS: not significantly different.

*Significantly different at 5% level.

**Significantly different at 1% level.

a, b, and c: indicating means within row differed significantly.

**Table 3:** Effect of fermented rice straw (FRSE) on VFA production (mmol), pH and IVDMD (%).

	Ethanol (control)	FRSE (10 mg)	FRSE (20 mg)	Significant
Acetate	36.09 ± 0.43^b^	38.67 ± 1.34^a^	38.65 ± 0.94^a^	**
Propionate	13.98 ± 0.42^ab^	14.19 ± 0.47^a^	13.55 ± 0.29^b^	*
Isobutyrate	0.75 ± 0 .01^a^	0.72 ± 0.01^b^	0.72 ± 0.01^b^	**
Butyrate	3.78 ± 0.12^b^	4.09 ± 0.07^a^	4.20 ± 0.06^a^	**
Isovalerate	1.61 ± 0.04^a^	1.51 ± 0.03^b^	1.49 ± 0.03^b^	**
Valerate	0.49 ± 0.01	0.51 ± 0.01	0.52 ± 0.01	**
Total	56.70 ± 0.69^b^	59.69 ± 1.62^a^	59.12 ± 1.12^a^	**
A/P	2.58 ± 0.09^c^	2.73 ± 0.10^b^	2.85 ± 0.07^a^	**
GP/VFA	1.31 ± 0.045^a^	1.19 ± 0.028^b^	1.13 ± 0.026^c^	**
CH_4_/VFA	0.14 ± 0.011^a^	0.12 ± 0.010^b^	0.10 ± 0.005^c^	**
pH	6.95 ± 0.01	6.97 ± 0.01	6.94 ± 0.03	NS
IVDMD	46.81 ± 0.84	46.36 ± 1.33	46.62 ± 1.47	NS

Data are mean ± SD.

NS: not significantly different.

*Significantly different at 5% level.

**Significantly different at 1% level.

a, b, and c: indicating means within row differed significantly.

**Table 4:** Effect of fermented rice straw extract (FRSE) on microbial population in the rumen liquid (cell/mL).

Microorganisms	Ethanol (control)	FRSE (10 mg)	FRSE (20 mg)	Significant
Total methanogens (×10^7^)	1.30 ± 0.172^a^	0.98 ± 0.121^b^	0.97 ± 0.248^b^	*
Methanobacteriales (×10^6^)	2.65 ± 0.215^a^	2.20 ± 0.177^b^	2.25 ± 0.240^b^	*
Anaerobic fungi (×10^6^)	2.69 ± 0.271^a^	1.78 ± 0.922^b^	0.47 ± 0.258^c^	**
Total bacteria (×10^11^)	2.55 ± 0.434^a^	2.38 ± 0.329^a^	1.93 ± 0.237^b^	*
*Ruminococcus albus* (×10^8^)	6.08 ± 0.966^b^	10.46 ± 0.668^a^	9.98 ± 1.139^a^	**
*Fibrobacter succinogenes* (×10^7^)	2.88 ± 0.742^b^	6.87 ± 1.840^a^	8.16 ± 0.686^a^	**
*Ruminococcus flavefaciens* (×10^6^)	1.05 ± 0.375^b^	1.46 ± 0.360^a^	1.31 ± 0.226^a^	*
Protozoa (×10^5^)	4.12 ± 0.893	4.65 ± 0.663	4.12 ± 0.692	NS

Data are mean ± SD.

NS: not significantly different.

*Significantly different at 5% level.

**Significantly different at 1% level.

a, b, and c: indicating means within row differed significantly.

**Table 5:** Comparative *in vitro* gas, methane and hydrogen productions by rumen microorganisms and rate of gas production between rice straw (RS) and fermented rice straw (FRS).

Gas production (mL)	RS	FRS	Significant
2 h	1.8 ± 0.3	3.2 ± 0.3	**
4 h	3.8 ± 0.8	5.5 ± 0.4	**
8 h	5.8 ± 1.1	7.4 ± 0.5	**
12 h	10.8 ± 1.7	10.5 ± 0.7	NS
24 h	31.9 ± 2.4	22.6 ± 2.1	**
36 h	47.0 ± 2.4	35.9 ± 2.9	**
48 h	55.9 ± 1.0	47.0 ± 2.2	**
Methane production			
%	11.26 ± 0.70	10.25 ± 0.51	*
*μ*M	28.15 ± 17.058	214.78 ± 9.087	**
H_2 _ production			
%	5.71 ± 0.36	5.15 ± 0.42	NS
*μ*M	141.41 ± 6.73	105.8 ± 7.77	*
Rate of GP (mL/h)	1.16 ± 0.020	0.979 ± 0.047	**
Rate of CH_4_ (mL/h)	0.13 ± 0.008	0.100 ± 0.004	**
CH_4_/total	0.11 ± 0.007	0.102 ± 0.005	*

Data are means ± SD.

NS: not significantly different.

*Significantly different at 5% level.

**Significantly different at 1% level.

**Table 6:** Effect of rice straw (RS) and fermented rice straw (FRS) on VFA production (mmol), pH and IVDMD (%).

	RS	FRS	Significant
Acetate	37.49 ± 2.22	34.92 ± 2.51	NS
Propionate	14.44 ± 0.75	13.01 ± 0.83	*
Isobutyrate	0.77 ± 0.04	0.76 ± 0.03	NS
Butyrate	3.80 ± 0.14	3.77 ± 0.16	NS
Isovalerate	1.66 ± 0.08	1.71 ± 0.08	NS
Valerate	0.49 ± 0.02	0.48 ± 0.02	NS
Total	58.65 ± 3.09	54.65 ± 3.55	NS
A/P	2.60 ± 0.09	2.68 ± 0.08	NS
GP/VFA	0.96 ± 0.059	0.86 ± 0.039	**
CH_4_/VFA	0.05 ± 0.004	0.04 ± 0.002	**
pH	6.87 ± 0.03	6.95 ± 0.04	**
IVDMD	45.81 ± 1.48	49.01 ± 0.79	**

Data are mean ± SD.

NS: not significantly different.

*Significantly different at 5% level.

**Significantly different at 1% level.

**Table 7:** Effect of rice straw (RS) and fermented rice straw (FRS) on microbial population in the rumen liquid (cell/mL).

Microorganisms	Treatment		Significant
	RS	FRS	
Total methanogens (×10^6^)	3.089 ± 0.553	2.34 ± 0.125	*
Methanobacteriales (×10^6^)	1.47 ± 0.154	0.96 ± 0.177	*
Anaerobic fungi (×10^6^)	10.79 ± 2.506	2.14 ± 0.666	*
Total bacteria (×10^11^)	2.86 ± 0.349	2.39 ± 0.276	**
*Ruminococcus albus* (×10^8^)	2.29 ± 0.126	5.40 ± 0.353	**
*Fibrobacter succinogenes* (×10^7^)	4.51 ± 0.922	1.82 ± 0.255	**
*Ruminococcus flavefaciens* (×10^6^)	0.93 ± 0.107	0.87 ± 0.110	NS
Protozoa (×10^5^)	8.50 ± 1.012	9.96 ± 1.407	NS

Data are mean ± SD.

NS: not significantly different.

*Significantly different at 5% level.

**Significantly different at 1% level.
